# Regional changes in intestinal permeability in cirrhosis are associated with mucosal bacteria

**DOI:** 10.1097/HC9.0000000000000221

**Published:** 2023-09-27

**Authors:** Patricia P. Bloom, Krishna Rao, Christine Bassis, Borko Nojkov, Vincent B. Young, Anna SF Lok

**Affiliations:** 1 Department of Internal Medicine, Division of Gastroenterology and Hepatology, University of Michigan, Ann Arbor, Michigan, USA; 2 Department of Internal Medicine, Division of Infectious Diseases, University of Michigan, Ann Arbor, Michigan, USA; 3 Department of Microbiology and Immunology, University of Michigan, Ann Arbor, Michigan, USA

## Abstract

**Background:**

Several complications of cirrhosis are theorized to result from the translocation of bacteria or their products across the intestinal epithelium. We aimed to assess epithelial permeability and associations with mucosal bacteria in patients with cirrhosis.

**Approach and Results:**

We collected 247 duodenum, ileum, and colon biopsies from 58 consecutive patients with cirrhosis and 33 controls during clinically indicated endoscopies. Patients with cirrhosis were similarly aged to controls (60 vs. 58 y) and had a median Model for End-stage Liver Disease of 8 (interquartile range 7, 10). Biopsies underwent 16S rRNA-encoding gene amplicon sequencing to determine mucosal bacteria composition and transepithelial electrical resistance (TEER) to determine epithelial permeability. In the entire cohort, there were regional differences in TEER with the lowest TEER (ie, more permeable) in the ileum; duodenum TEER was 43% higher and colon TEER 20% higher than ileum TEER (ANOVA *p* = 0.0004). When comparing patients with cirrhosis and controls, both TEER (26% lower in cirrhosis, *p* = 0.006) and alpha diversity differed in the duodenum (27% lower in cirrhosis, *p* = 0.01) but not ileum or colon. A beta-binomial model found that 26 bacteria were significantly associated with TEER. *Bifidobacteriaceae Bifidobacterium* in duodenal mucosa was protective of epithelial permeability and future hospitalization for hepatic decompensation.

**Conclusions:**

Duodenal epithelial permeability was higher, and mucosal bacteria alpha diversity was lower in cirrhosis compared to controls, while no such differences were seen in the ileum or colon. Specific bacteria were associated with epithelial permeability and future hepatic decompensation.

## Graphical abstract

**Figure:**
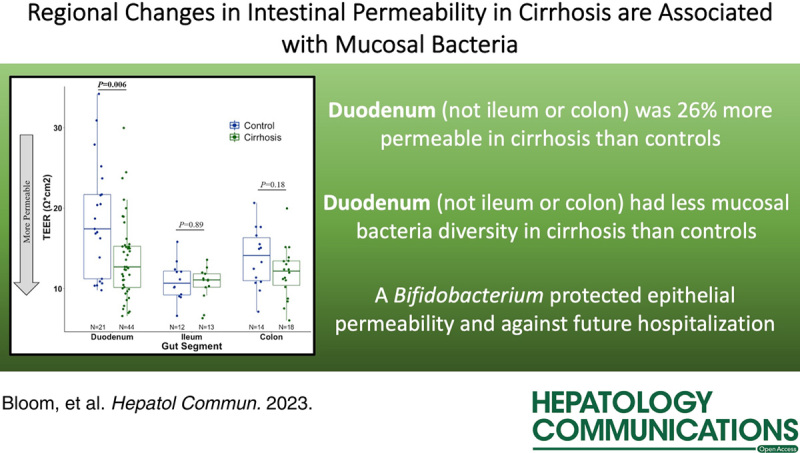

## BACKGROUND

Several complications of cirrhosis are thought to result from increased intestinal permeability or “leaky gut.” The translocation of bacteria or their products across the intestinal epithelium leads to systemic inflammation and downstream complications. Prior work demonstrates increased duodenal^1–3^ and colonic epithelial permeability^4^ in compensated cirrhosis; however, many questions still remain regarding regional differences in epithelial permeability along the intestinal tract in cirrhosis or how this permeability evolves from compensated to decompensated cirrhosis. Interventions targeting the small intestine differ from those targeting the colon; therefore, isolating the most permeable gut segment(s) in cirrhosis will focus future research on the most important therapeutic targets.

One such knowledge gap pertains to the relationship between intestinal permeability and mucosal microbiota in cirrhosis. Certain bacteria may have a protective effect on gut barrier function through the production of molecules that support epithelial health,^5^ while other bacteria may have a detrimental effect through immune modulation.^6^ We have previously shown in a pilot study of 24 patients with compensated cirrhosis that particular duodenal mucosal bacteria are associated with duodenal permeability.^1^ In the present study, we enrolled a larger number of patients with cirrhosis, including compensated and decompensated disease, to study epithelial permeability in 3 gut segments (duodenum, ileum, and colon). We aimed to determine in cirrhosis which gut segment is most permeable and to evaluate the association between mucosal bacteria and epithelial permeability. In addition, we evaluated if mucosal bacteria composition predicted future hepatic decompensation.

## METHODS

### Study design

We conducted a prospective study, collecting clinical, mucosal bacteria composition, and intestinal permeability data from patients at a baseline endoscopy. From October 2020 to September 2022, we collected duodenal aspirate, duodenum, ileum, and colon tissue biopsies from patients with cirrhosis and controls undergoing routine outpatient upper endoscopy and/or colonoscopy. The clinical indication for upper endoscopy in patients with cirrhosis was esophageal variceal screening. The principal clinical indications for upper endoscopy in controls were globus sensation, dysphagia, anemia without iron deficiency, and prebariatric surgery. The clinical indication for colonoscopy in all patients was colorectal cancer screening. This study was approved by the University of Michigan Institutional Review Board, and all patients provided informed written consent for the study. The majority of the methods for this study were previously published.^1^ All research was conducted in accordance with both the Declarations of Helsinki and Istanbul.

### Patient selection

Only patients without overt HE and with the capacity to consent were enrolled. Patients and controls were excluded if they had inflammatory bowel disease or used a nonrifaximin antibiotic (patients on rifaximin could be included) or immunosuppressive medications in the preceding 4 weeks. Patients who had received chemotherapy or systemic hepatocellular carcinoma treatment in the prior year were excluded. Patients with a platelet count <50/nL or international normalized ratio >1.5 were excluded to minimize bleeding risk. Cirrhosis was diagnosed by a hepatologist based on biopsy, imaging, elastography, or a history of hepatic decompensation. Controls had no evidence of liver disease and no gastrointestinal symptoms. To minimize confounding effects of age and medical comorbidities on microbiota composition, we enrolled controls with similar age and the number of nonhepatic comorbidities as patients with cirrhosis. We also included data from a previously published cohort of 10 controls, and collected and analyzed with the same protocols and instruments as described.^7^ Controls were heterogeneous in terms of nonhepatic comorbidities.

### Clinical data collection

Both controls and patients with cirrhosis were asked about proton pump inhibitors, lactulose use, antibiotic, and probiotic use prior to the endoscopy. Other clinical data, including age, sex, and comorbidities, were obtained from chart review. An extrahepatic Charlson Comorbidity Index was calculated for every patient by subtracting 3 points for advanced liver disease for cirrhosis patients (subtraction not completed for controls as they had no liver disease). Additional cirrhosis-related information was collected from patients with cirrhosis including the Model for End-stage Liver Disease (MELD) score, Child-Pugh score, comorbidities, and etiology of liver disease. Cirrhosis patients were followed through chart review until death, liver transplantation, or hospitalization for hepatic decompensation. Clinic notes, and telephone and patient portal messages were reviewed to identify hospitalizations outside the University of Michigan network; however, the majority of these patients had all their care at the University of Michigan.

### Microbiome assessment

One biopsy sample per segment per patient was analyzed for mucosal microbiota composition through 16S rRNA-encoding gene amplicon sequence analysis. Mucosal biopsies underwent DNA isolation using a Qiagen MagAttract Powermag microbiome DNA isolation kit (Germantown, MD). Barcoded dual-index primers specific to the V4 region of the 16S rRNA gene were used to amplify DNA, using a “touchdown PCR” protocol given that duodenal mucosa is potentially low biomass.^8^ Multiple negative controls were run in parallel. Microbiome sequencing occurred in three separate runs. Libraries were prepared and sequenced using the Illumina 500-cycle MiSeq reagent kit V2 (San Diego, CA).^9^ The mothur software package v1.48.0 was used to trim, screen, and align sequences, calculate distances, remove chimeras, assign sequences with ≥97% similarity to operational taxonomic units, and calculate alpha and beta diversity.^9,10^ Taxonomic classification was based on Ribosomal Database Project (version 18, 6/2020).^11^ The SILVA rRNA reference alignment (release v132) was used to align the V4 region.^12^ The overall sequencing error rate, based on comparison to a mock community, was 0.026%. Microbiome sequencing data has been submitted to the NIH Sequence Read Archive (Submission number: SUB13657574).

### Epithelial permeability assessment

Two mucosal biopsies per segment per patient were evaluated for epithelial permeability through transepithelial electrical resistance (TEER), a measure of paracellular permeability. After temperature and pH stabilization, duodenal biopsies underwent TEER analysis within 30 minutes of the collection with a micro-Snapwell system with an Endohm sural sensory nerve action potential electrode attached to an EVOM2 epithelial volt-ohm meter (World Precision Instruments), following published protocols.^7^ Results were reported as an average of the 2 biopsies and expressed in ohms times centimeter squared (Ωcm^2^).

### Statistical analysis

Descriptive statistics were reported as median and interquartile range (IQR) for continuous variables, and the number and percentages for categorical variables. Wilcoxon rank sum tests were used to compare continuous variables between 2 groups and the Fisher exact test to compare 2 categorical variables. Linear regression was used to identify covariates that influenced TEER. With regard to microbiome data analysis, alpha diversity was assessed with inverse Simpson, as generated in mothur.^10^ Comparisons of microbiota community structure between groups were based on analysis of molecular variance (AMOVA) of the Yue and Clayton dissimilarity index (θ_YC_) and displayed as principal coordinates analysis of θ_YC_ distances.^13^ Beta-binomial regression models were used to associate the relative abundance of individual taxa with TEER and patient type (cirrhosis vs. controls). Unlike other modeling techniques, beta-binomial models allow for jointly assessing the relative abundance and differential variability, the latter of which is valuable because some disease states manifest increased variability in bacterial abundance.^14^ Cox regression was used to evaluate differences in time to hospitalization for hepatic decompensation based on the presence versus absence of bacterial taxa identified in the beta-binomial model to be associated with TEER, censoring for death, liver transplantation, nondecompensation hospitalizations, and loss to follow-up. Survival analyses were displayed as Kaplan-Meier curves. R packages were used to analyze mothur outputs and other data, and for figure creation (R Foundation for Statistical Computing, Vienna, Austria).^15^

Based on prior work showing reduced TEER in patients with cirrhosis compared to controls, 17.0 (SD 0.8) versus 21.4 (SD 1.1) Ωcm^2^,^3^ a sample size of 10 samples per gut segment per patient group would be sufficient to provide 90% power, with an alpha of 0.05. Due to a lack of available data about duodenal mucosal microbiota composition, we were unable to base power calculations on microbiome feature differences. Therefore, a larger cohort was enrolled to increase the power of additional microbiome analyses.

## RESULTS

We collected 247 mucosal biopsies and 46 duodenal aspirates from 58 patients with cirrhosis and 33 controls. Of the mucosal biopsies, 122/247 were used to analyze TEER, and 125/247 were used for 16S rRNA-encoding gene amplicon sequencing. Biopsies were obtained from the following locations: 74 duodenums, 33 ileums, and 38 colons (Supplemental Table S1, http://links.lww.com/HC9/A419). Patients with cirrhosis were similarly aged as controls (60 vs. 58 y; *p* = 0.68) and had a similar number of extrahepatic comorbidities (2 versus 2; *p* = 0.35; Table 1). Controls were more likely to be female (61% vs. 38%, *p* = 0.03) and less likely to use lactulose (3% vs. 28%, *p* = 0.004) or rifaximin (0% vs. 19%, *p* = 0.006) than patients with cirrhosis. Patients with cirrhosis had a median MELD of 8 (IQR 7, 10). The indications for upper endoscopy in controls were dysphagia (8), globus sensation (7), anemia (2), prebariatric surgery (2), fatigue (1), and cancer screening (1).

**TABLE 1:** Clinical Characteristics^a^

	Cirrhosis (N = 58 patients)	Controls (N = 33 patients)
Procedure type, n (%)		
Upper endoscopy only	33 (57)	13 (39)
Colonoscopy only	1 (2)	12 (36)
Both procedures	24 (41)	8 (24)
Demographics		
Age (y)	60 (53, 65)	58 (49, 68)
Female sex, n (%)	22 (38)	20 (61)
Extrahepatic Charlson Comorbidity Index	2 (1, 4)	2 (1, 4)
Proton pump inhibitor, n (%)	11 (19)	10 (30)
Probiotic, n (%)	10 (17)	6 (18)
Lactulose^b^, n (%)	16 (28)	1 (3)
Rifaximin, n (%)	11 (19)	0
MELD, n (%)	8 (7, 10)	NA
Child-Pugh Class A, n (%)	30 (52)	NA
Child-Pugh Class B, n (%)	26 (45)	NA
Child-Pugh Class C, n (%)	2 (3)	NA
NAFLD, n (%)	30 (52)	NA
Alcohol-associated, n (%)	23 (40)	NA
Viral hepatitis B or C, n (%)	6 (10)	NA
Gastroesophageal varices, n (%)	42 (74)	NA
Ascites, n (%)	28 (49)	NA
HE, n (%)	16 (28)	NA
HCC, n (%)	6 (10)	NA

a n (%); Median (interquartile range).

b One control was prescribed lactulose for constipation, while patients with cirrhosis were principally prescribed lactulose for the history of HE.

Abbreviation: MELD, Model for End-Stage Liver Disease.

### Intestinal permeability in cirrhosis versus controls

**FIGURE 1:**
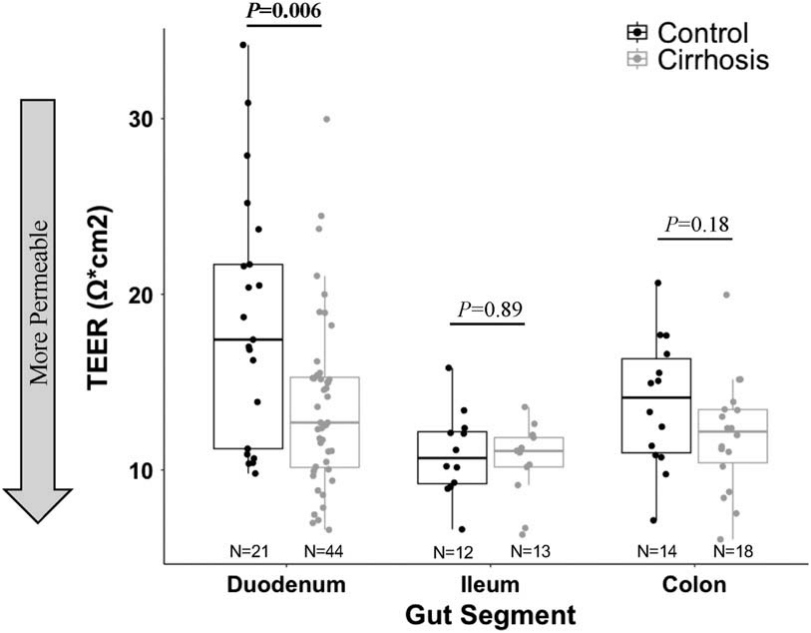
Intestinal permeability by gut segment and patient type. Transelectrical epithelial resistance (TEER) compared between patients with cirrhosis and controls by gut segment. Lower TEER signifies a more permeable epithelial layer, while higher TEER signifies a less permeable epithelial layer.

In the entire cohort, TEER varied by gut segment (ANOVA *p* = 0.0004) with ileum having the lowest TEER (ie, most permeable; duodenum: 15.3 Ωcm^2^, ileum: 10.7 Ωcm^2^, and colon: 12.8 Ωcm^2^). These findings persisted in separate analyses of patients with cirrhosis (ANOVA *p* = 0.046) and controls (ANOVA *p* = 0.0008). Duodenum was the only gut segment in which TEER differed between patients with cirrhosis and controls (13.7 ± 5.0 Ωcm^2^ vs. 18.5 ± 7.1 Ωcm^2^; *p* = 0.006, Figure 1).

TEER in the entire cohort and all gut segments did not correlate with age (Pearson *r* = 0.02 and *p* = 0.87). In addition, the duodenum remained more permeable in patients with cirrhosis than controls when excluding the 19 patients on proton pump inhibitors (*p* = 0.02), the 12 patients on lactulose (*p* = 0.0006), the 10 patients on rifaximin (*p* = 0.002), and the 26 patients who underwent bowel purge for colonoscopy (*p* = 0.008). Among patients with cirrhosis, those with alcohol etiology showed a trend toward lower duodenal TEER (12.3 ± 3.5 Ωcm^2^ vs. 14.7 ± 5.6 Ωcm^2^; *p* = 0.10), with no difference in the ileum or colonic TEER, compared to those with other etiologies. Within patients with cirrhosis, no gut segment differed in TEER by the history of ascites, HE, or by MELD (≥10 vs. < 10; Supplemental Table S2, http://links.lww.com/HC9/A420). Finally, in a comparison of the 10 patients with cirrhosis and 5 controls who underwent sampling from all 3 gut segments, the ileum remained the segment with numerically lowest TEER, and duodenal TEER was numerically lower in patients with cirrhosis than controls.

### Bacteria composition varies by patient type and gut segment

**FIGURE 2:**
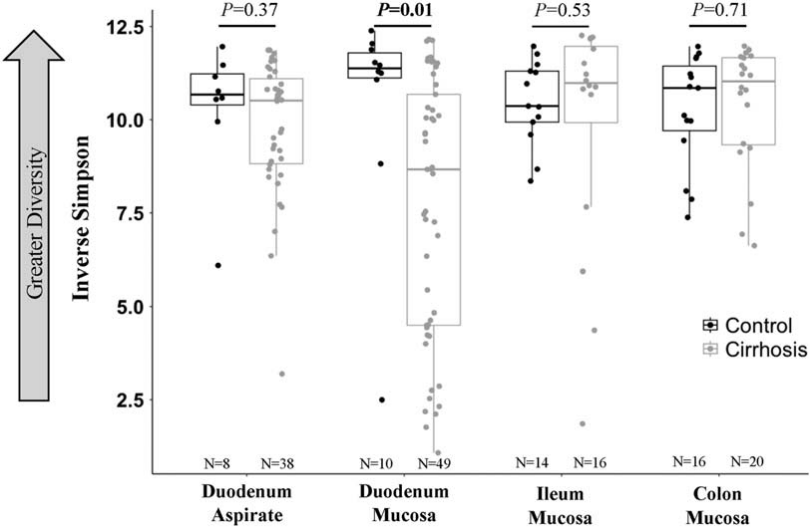
Alpha diversity by gut segment and patient type. Alpha diversity, as measured by inverse Simpson, compared between patients with cirrhosis and controls, by sample location. Lower inverse Simpson signifies lower alpha diversity.

The mucosal bacteria had lower alpha diversity in cirrhosis than in controls for all gut segments combined (inverse Simpson 10.4 vs. 8.7, *p* = 0.03), and separately in duodenal biopsies (inverse Simpson 10.4 vs. 7.6, *p* = 0.01) but not the ileal or colonic biopsies or duodenal aspirates (Figure 2). Patients with cirrhosis had a distinct mucosal bacteria community structure relative to controls in all gut segments combined based on AMOVA of the θ_YC_ dissimilarity index (*p* < 0.001; Supplemental Table S3, http://links.lww.com/HC9/A421), and 40 taxa differed between the groups based on LEfSe. Patients with cirrhosis also had a distinct mucosal bacterial community in duodenal aspirates relative to controls (*p* = 0.011).

**FIGURE 3:**
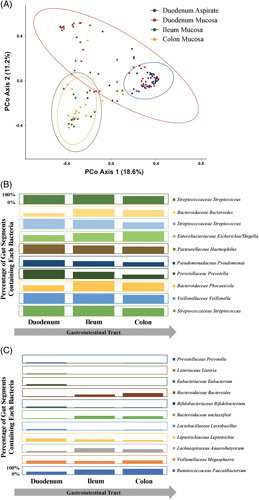
Within cirrhosis, mucosal bacteria varied by gut segment. (A) Principal coordinate analysis of θ_YC_ distances comparing bacterial community composition sampling sites (duodenum aspirate, duodenum mucosa, ileum mucosa, and colon mucosa) in patients with cirrhosis. (B) Shift in the presence of the 10 most abundant bacteria across gut segments in cirrhosis. Each row represents a single bacterium. Each column represents a gut segment (duodenum, ileum, and colon). Each box represents the percentage of gut segments containing that bacteria. For example, the box in the upper right corner of the figure indicates that 80% of patients with cirrhosis had *Streptococcaceae Streptococcus* present in their colon mucosa compared to 100% in the ileum and 98% in the duodenum. (C) Shift in the presence of the 11 bacteria associated with duodenal TEER in cirrhosis. Each row represents a single bacterium. Each column represents a gut segment (duodenum, ileum, and colon). Each box represents the percentage of gut segments containing that bacteria. For example, the box in the bottom right corner of the figure indicates that 70% of patients with cirrhosis had *Ruminococcaceae Faecalibacterium* present in their colons compared to 63% in ileums and 35% in duodenums.

Among patients with cirrhosis, mucosal bacteria composition varied by gut segment (*p* < 0.001 in AMOVA; Figure 3A). Bacteria composition in duodenum mucosa differed from ileum and colon mucosa (both *p* < 0.001 in AMOVA), while ileum and colon mucosal bacteria compositions were similar (*p* = 0.998). Duodenal aspirate bacteria composition also differed from duodenal, ileum, and colon mucosa (all *p* < 0.001). The 10 most abundant bacteria shifted slightly in relative abundance across gut segments (Figure 3B). For example, *Streptococcaceae* was more likely to be present in the duodenal mucosa than other gut segments, but 2 *Bacteroidaceae* were less likely to be present in the duodenum. Alpha diversity of duodenal mucosa (inverse Simpson, no purge: 7.4 vs. purge: 8.1, *p* = 0.62) and duodenal mucosa bacterial community structure (AMOVA, *p* = 0.39) did not differ between those with and without bowel purge for colonoscopy in patients with cirrhosis. All ileum and colon samples were taken from patients who had undergone bowel purges.

### Mucosal bacteria are associated with intestinal permeability in cirrhosis

**FIGURE 4:**
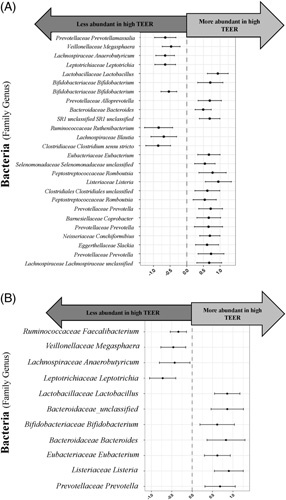
Mucosal bacteria associated with intestinal permeability in cirrhosis. (A) In all gut segments combined, 26 bacteria were significantly associated with TEER in a beta-binomial model with a false discovery rate of 0.05. (B) Eleven duodenal bacteria were significantly associated with duodenal TEER in a beta-binomial model with a false discovery rate of 0.05.

A beta-binomial model found 26 bacteria significantly associated with TEER, in all gut segments combined, with a false discovery rate of 0.05 (Figure 4A). A beta-binomial model focused on the duodenum found 11 bacteria significantly associated with TEER (Figure 4B). For example, *Bifidobacteriaceae Bifidobacterium*, *Lactobacillaceae Lactobacillus*, and 2 *Bacteroidaceae* bacteria were protective of TEER (less permeable). The presence of most of these 11 bacteria in the duodenum is markedly different from that in the ileum and colon (Figure 3C).

### Mucosal bacteria associated with future hospitalizations for hepatic decompensation and HE

In a follow-up of a median of 192 days (IQR 79, 548), 14 patients were hospitalized for cirrhosis decompensation after a median of 90 days (IQR 50, 173), 7 of which were due to overt HE (2 of these patients had prior HE and 5 did not).

**FIGURE 5:**
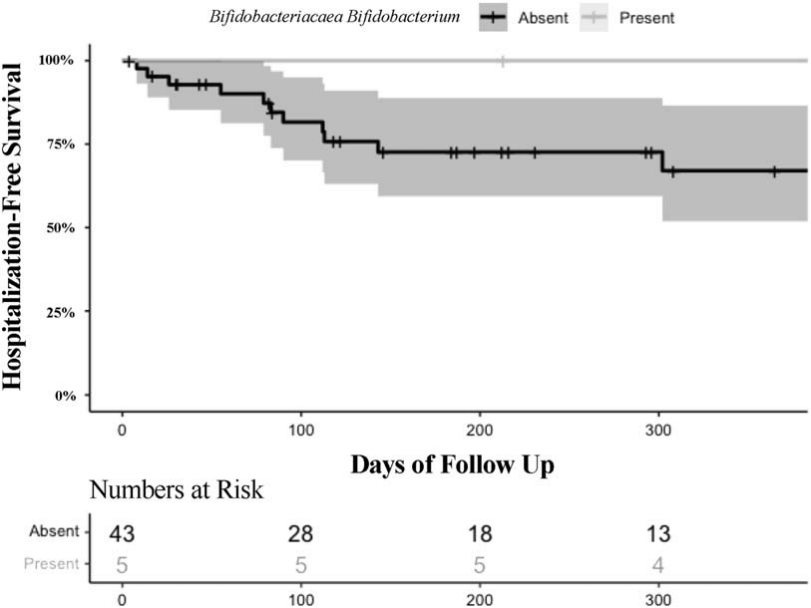
Impact of *Bifidobacteriaceae Bifidobacterium* on time to hospitalization for cirrhosis decompensation. Kaplan-Meier curve demonstrating time free of hospitalization for hepatic decompensation in those with (present) and without (absent) *Bifidobacteriaceae Bifidobacterium* in their duodenal mucosa. Patients were censored for death, hospitalizations for nonhepatic reasons or loss of follow-up.

When we limited our analyses to the 11 duodenal taxa associated with TEER, the absence of *Bifidobacteriaceae Bifidobacterium* in duodenal mucosa was associated with significantly higher rates of hospitalization for cirrhosis decompensation (Figure 5 includes 95% CI bands). Of the 43 patients without *Bifidobacteriaceae Bifidobacterium* in their duodenal mucosa, 15 (35%) experienced hospitalization for cirrhosis decompensation by 90 days and 21 (49%) by 180 days postbiopsy, while none of the 5 patients with *Bifidobacteriaceae Bifidobacterium* in their duodenal mucosa experienced hospitalization for cirrhosis decompensation after median 231 days of follow-up (IQR 122, 589). None of the 11 taxa predicted the time to overt HE hospitalization though the number of events was small.

## DISCUSSION

In this study, we compared regional variation in intestinal permeability along the gastrointestinal tract between patients with cirrhosis and controls with no liver disease and investigated associations between mucosal bacteria composition, epithelial permeability, and cirrhosis outcomes. Through direct measurements, this work sidesteps the uncertainty inherent to more limited, indirect techniques used to assess gut mucosal bacteria (stool samples) and intestinal permeability (serum lipopolysaccharide). We found that the duodenum was the only gut segment more permeable and with lower bacterial diversity in patients with cirrhosis than in controls. We, furthermore, found that certain mucosal bacteria were associated with epithelial permeability and also predicted future hospitalization for hepatic decompensation.

This study supports and expands on prior work by confirming regional variation in epithelial permeability along the gastrointestinal tract. Our study measured TEER, which has been validated and widely used to evaluate functional paracellular epithelial permeability, dominantly regulated by tight junction proteins that connect the apical junctions of epithelial cells.^16–19^ Paracellular permeability is just 1 epithelial permeability pathway, distinct, for example, from apoptotic injury of mucosal ulceration where transepithelial permeability is prominent.^20^ A review primarily of animal models demonstrates that the small bowel is more permeable than the colon due to the differential expression of tight junction proteins, as well as the villous and crypt architecture of the small bowel epithelium.^21^ This study found the ileum to be the most permeable gut segment, more so than the colon, which fits with prior research. This study also supports prior work by confirming that gut bacterial composition differs between patients with cirrhosis and controls without liver disease. Much of the prior work demonstrating this difference is based on fecal samples alone.^22–24^ A few studies have demonstrated a difference in sigmoid mucosal bacterial composition between cirrhosis and control populations,^25,26^ but this is the first study to examine mucosal bacteria in the duodenum, ileum, and ascending colon in patients with cirrhosis and controls.

The primary finding of this work is that the duodenum was the only gut segment with greater epithelial permeability and lower bacterial alpha diversity in cirrhosis compared to controls with no liver disease. These differences in permeability and bacteria composition did not appear to be driven by confounders such as comorbidities or age, as patients with cirrhosis and controls were well-matched. Duodenal TEER was lower in patients with cirrhosis, even when excluding patients on proton pump inhibitors, lactulose, or rifaximin. Also, TEER did not correlate with age. Prior work suggested that portal hypertension influences intestinal permeability in cirrhosis.^27^ Portal hypertensive changes to intestinal mucosa are more common and severe in the upper gastrointestinal than lower gastrointestinal tract; thus, portal hypertensive changes may explain why the duodenum in cirrhosis was more permeable. Among patients with cirrhosis, duodenal mucosa also contained a unique set of bacteria, differing from duodenal aspirate, ileal mucosa, and colonic mucosa. For example, *Streptococcaceae* were more likely to be present in the duodenal mucosa than other gut segments, but 2 *Bacteroidaceae* were less likely to be present in the duodenum. While the colon has been the focus of prior cirrhosis microbiome investigation, this study highlights the duodenum as uniquely differentiating patients with cirrhosis and controls without liver disease.

Both control and cirrhosis groups demonstrated a wide range in duodenal epithelial permeability. We examined whether this heterogeneity was related to mucosal bacteria composition. We found that certain mucosal bacteria are protective of epithelial permeability, while other bacteria are associated with high permeability (or leaky gut). *Veillonellaceae* and *Leptotrichiaceae* bacteria were associated with high epithelial permeability in all gut segments combined, as well as specifically in the duodenum. *Veillonella* are opportunistic pathogens, previously associated with several chronic diseases and increased intestinal permeability and gut translocation in an animal model.^28–30^ Some *Veillonella* strains contain sialidase that can break down the mucin layer and increase gut permeability.^31^ These *Veillonella* are depleted by rifaximin, which may reduce systemic inflammation and contribute to rifaximin’s therapeutic mechanism.^31^ *Leptotrichiaceae*, generally of oral origin, have also been associated with chronic periodontitis and separately Lynch syndrome.^32,33^ On the other hand, *Bifidobacteriaceae Bifidobacterium*, *Lactobacillaceae Lactobacillus*, and *Bacteroidaceae Bacteroides* bacteria were protective of the intestinal mucosal barrier in all gut segments combined, as well as specifically in the duodenum. Certain *Lactobacillus* and *Bifidobacterium* strains increase tight junction protein production and produce short-chain fatty acids—an important nutritional source for intestinal epithelia.^34–39^ While the majority of short-chain fatty acids are produced in the colon, they are also produced in the small bowel^40,41^ and have a protective effect on duodenal permeability in an animal model.^42,43^

A striking finding in this study was that *Bifidobacteriaceae Bifidobacterium* in the duodenal mucosa of patients with cirrhosis was protective of future hospitalization for liver decompensation. It is possible that this decrease in liver-related hospitalization is mediated by an improvement in barrier function and diminished intestinal permeability induced by *Bifidobacteriaceae Bifidobacterium*.

The findings of this study are limited by factors inherent to the study design. First, the entire extent of the gastrointestinal tract could not be sampled. Due to safety and feasibility restrictions, we were unable to sample the jejunum. Second, the cirrhosis population investigated in this study was skewed toward mild and compensated diseases, as evidenced by a median MELD of 8 and median platelet count of 129/nL, because of the exclusion of patients with platelet count <50/nL or international normalized ratio >1.5 to minimize bleeding risk. In addition, patients on nonrifaximin antibiotics were excluded, so as not to confound bacteria analyses; however, this excluded patients with a history of spontaneous bacterial peritonitis and other recent infections. Thus, while 48% of the cirrhosis sample had Child-Pugh B or C cirrhosis, their decompensations were overall mild with ascites medically managed with diuretics or HE well-managed by lactulose. Third, to diminish bias, we enrolled consecutive patients who met inclusion criteria but, as such, enrolled a mix of patients undergoing upper endoscopy or colonoscopy alone. Although only 15 participants had samples from all 3 gut segments for direct comparison, the findings in this subgroup were consistent. Finally, confounders are a challenge in studying patients with decompensated cirrhosis. For example, patients with cirrhosis in this study were on rifaximin (19%), lactulose (28%), and proton pump inhibitors (19%), all of which may influence intestinal bacteria composition. However, it is impossible to enroll sufficient patients with decompensated cirrhosis not on any of these medications, and the exclusion of patients on these medications would prevent the generalizability of the results.

In conclusion, patients with cirrhosis exhibit regional variation in intestinal permeability along the gastrointestinal tract. The duodenum in particular is more permeable and has lower bacterial diversity in cirrhosis compared to controls with matched age and comorbidities. Furthermore, certain mucosal bacteria are associated with epithelial permeability and may predict future hospitalization for hepatic decompensation. Future prospective research will explore the potential causative role of microbiota in influencing intestinal permeability in cirrhosis, including patients with greater decompensation. Additional investigations to understand why the duodenum is more permeable in cirrhosis, with specific studies into portal hypertension, tight junction protein composition and distribution, and mucosal bacterial function, are also necessary. The important implication of this work is the potential to target mucosal bacteria as a means to impact the intestinal barrier and cirrhosis complications.

## Supplementary Material

## References

[1] Bloom PP Rao K Bassis CM Zhou SY Nojkov B Owyang C . Duodenal permeability is associated with mucosal microbiota in compensated cirrhosis. Clin Transl Gastroenterol. 2022;13:e00522. 36000993 10.14309/ctg.0000000000000522 PMC9624490

[2] Assimakopoulos SF Tsamandas AC Tsiaoussis GI Karatza E Triantos C Vagianos CE . Altered intestinal tight junctions’ expression in patients with liver cirrhosis: A pathogenetic mechanism of intestinal hyperpermeability. Eur J Clin Invest. 2012;42:439–46. 22023490 10.1111/j.1365-2362.2011.02609.x

[3] Du Plessis J Vanheel H Janssen CEI Roos L Slavik T Stivaktas PI . Activated intestinal macrophages in patients with cirrhosis release NO and IL-6 that may disrupt intestinal barrier function. J Hepatol. 2013;58:1125–32. 23402745 10.1016/j.jhep.2013.01.038

[4] Pijls KE Koek GH Elamin EE de Vries H Masclee AAM Jonkers DMAE . Large intestine permeability is increased in patients with compensated liver cirrhosis. Am J Physiol Gastrointest Liver Physiol. 2014;306:G147–53. 24264047 10.1152/ajpgi.00330.2013

[5] Sanders ME Merenstein DJ Reid G Gibson GR Rastall RA . Probiotics and prebiotics in intestinal health and disease: From biology to the clinic. Nat Rev Gastroenterol Hepatol. 2019;16:605–16. 31296969 10.1038/s41575-019-0173-3

[6] de Vos WM Tilg H Van Hul M Cani PD . Gut microbiome and health: mechanistic insights. Gut. 2022;71:1020–32. 35105664 10.1136/gutjnl-2021-326789 PMC8995832

[7] Nojkov B Zhou SY Dolan RD Davis EM Appelman HD Guo X . Evidence of duodenal epithelial barrier impairment and increased pyroptosis in patients with functional dyspepsia on confocal laser endomicroscopy and “ex vivo” mucosa analysis. Am J Gastroenterol. 2020;115:1891–901. 33156108 10.14309/ajg.0000000000000827 PMC8409129

[8] Koenigsknecht MJ Theriot CM Bergin IL Schumacher CA Schloss PD Young VB . Dynamics and establishment of Clostridium difficile infection in the murine gastrointestinal tract. Infect Immun. 2015;83:934–41. 25534943 10.1128/IAI.02768-14 PMC4333439

[9] Kozich JJ Westcott SL Baxter NT Highlander SK Schloss PD . Development of a dual-index sequencing strategy and curation pipeline for analyzing amplicon sequence data on the MiSeq Illumina sequencing platform. Appl Environ Microbiol. 2013;79:5112–20. 23793624 10.1128/AEM.01043-13 PMC3753973

[10] Schloss PD Westcott SL Ryabin T Hall JR Hartmann M Hollister EB . Introducing mothur: Open-source, platform-independent, community-supported software for describing and comparing microbial communities. Appl Environ Microbiol. 2009;75:7537–41. 19801464 10.1128/AEM.01541-09 PMC2786419

[11] Cole JR Wang Q Fish JA Chai B McGarrell DM Sun Y . Ribosomal Database Project: Data and tools for high throughput rRNA analysis. Nucleic Acids Res. 2014;42(Database issue):D633–42. 24288368 10.1093/nar/gkt1244 PMC3965039

[12] Quast C Pruesse E Yilmaz P Gerken J Schweer T Yarza P . The SILVA ribosomal RNA gene database project: improved data processing and web-based tools. Nucleic Acids Res. 2013;41(Database issue):D590–6. 23193283 10.1093/nar/gks1219 PMC3531112

[13] Yue JC CM . A similarity measure based on species proportions. Commun Stat-Theory Methods. 2005;34:2123–31.

[14] Martin BD Witten D Willis AD . Modeling microbial abundances and dysbiosis with beta-binomial regression. Ann Appl Stat. 2020;14:94–115. 32983313 10.1214/19-aoas1283 PMC7514055

[15] R Core Team (2020). R: A language and environment for statistical computing. R Foundation for Statistical Computing, V., Austria. Accessed October 1, 2022. https://www.R-project.org/.

[16] Srinivasan B Kolli AR Esch MB Abaci HE Shuler ML Hickman JJ . TEER measurement techniques for in vitro barrier model systems. J Lab Autom. 2015;20:107–126. 25586998 10.1177/2211068214561025 PMC4652793

[17] Madara JL Barenberg D Carlson S . Effects of cytochalasin D on occluding junctions of intestinal absorptive cells: Further evidence that the cytoskeleton may influence paracellular permeability and junctional charge selectivity. J Cell Biol. 1986;102:2125–36. 3711143 10.1083/jcb.102.6.2125 PMC2114240

[18] Singh P Grabauskas G Zhou SY Gao J Zhang Y Owyang C . High FODMAP diet causes barrier loss via lipopolysaccharide-mediated mast cell activation. JCI Insight. 2021;6:e146529. 34618688 10.1172/jci.insight.146529 PMC8663790

[19] Vanheel H Vicario M Vanuytsel T Van Oudenhove L Martinez C Keita ÅV . Impaired duodenal mucosal integrity and low-grade inflammation in functional dyspepsia. Gut. 2014;63:262–71. 23474421 10.1136/gutjnl-2012-303857

[20] Camilleri M . Leaky gut: mechanisms, measurement and clinical implications in humans. Gut. 2019;68:1516–26. 31076401 10.1136/gutjnl-2019-318427 PMC6790068

[21] Camilleri M Madsen K Spiller R Van Meerveld BG Verne GN . Intestinal barrier function in health and gastrointestinal disease. Neurogastroenterol Motil. 2012;24:503–12. 22583600 10.1111/j.1365-2982.2012.01921.x PMC5595063

[22] Qin N Yang F Li A Prifti E Chen Y Shao L . Alterations of the human gut microbiome in liver cirrhosis. Nature. 2014;513:59–64. 25079328 10.1038/nature13568

[23] Solé C Guilly S Da Silva K Llopis M Le-Chatelier E Huelin P . Alterations in gut microbiome in cirrhosis as assessed by quantitative metagenomics: Relationship with acute-on-chronic liver failure and prognosis. Gastroenterology. 2021;160:206–18.e13. 32941879 10.1053/j.gastro.2020.08.054

[24] Bajaj JS Heuman DM Hylemon PB Sanyal AJ White MB Monteith P . Altered profile of human gut microbiome is associated with cirrhosis and its complications. J Hepatol. 2014;60:940–7. 24374295 10.1016/j.jhep.2013.12.019 PMC3995845

[25] Bajaj JS Hylemon PB Ridlon JM Heuman DM Daita K White MB . Colonic mucosal microbiome differs from stool microbiome in cirrhosis and hepatic encephalopathy and is linked to cognition and inflammation. Am J Physiol Gastrointest Liver Physiol. 2012;303:G675–85. 22821944 10.1152/ajpgi.00152.2012 PMC3468538

[26] Bajaj JS Betrapally NS Hylemon PB Thacker LR Daita K Kang DJ . Gut microbiota alterations can predict hospitalizations in cirrhosis independent of diabetes mellitus. Sci Rep. 2015;5:18559. 26692421 10.1038/srep18559 PMC4686976

[27] Reiberger T Ferlitsch A Payer BA Mandorfer M Heinisch BB Hayden H . Non-selective betablocker therapy decreases intestinal permeability and serum levels of LBP and IL-6 in patients with cirrhosis. J Hepatol. 2013;58:911–21. 23262249 10.1016/j.jhep.2012.12.011

[28] Chen X Li P Liu M Zheng H He Y Chen MX . Gut dysbiosis induces the development of pre-eclampsia through bacterial translocation. Gut. 2020;69:513–22. 31900289 10.1136/gutjnl-2019-319101

[29] De Cruz P Kang S Wagner J Buckley M Sim WH Prideaux L . Association between specific mucosa-associated microbiota in Crohn’s disease at the time of resection and subsequent disease recurrence: A pilot study. J Gastroenterol Hepatol. 2015;30:268–78. 25087692 10.1111/jgh.12694

[30] Kummen M Holm K Anmarkrud JA Nygård S Vesterhus M Høivik ML . The gut microbial profile in patients with primary sclerosing cholangitis is distinct from patients with ulcerative colitis without biliary disease and healthy controls. Gut. 2017;66:611–9. 26887816 10.1136/gutjnl-2015-310500

[31] Patel VC Lee S McPhail MJW Da Silva K Guilly S Zamalloa A . Rifaximin-α reduces gut-derived inflammation and mucin degradation in cirrhosis and encephalopathy: RIFSYS randomised controlled trial. J Hepatol. 2022;76:332–42. 34571050 10.1016/j.jhep.2021.09.010

[32] Ferrarese R Zuppardo RA Puzzono M Mannucci A Amato V Ditonno I . Oral and fecal microbiota in lynch syndrome. J Clin Med. 2020;9:2735. 32847083 10.3390/jcm9092735 PMC7563889

[33] Balmasova IP Olekhnovich EI Klimina KM Korenkova AA Vakhitova MT Babaev EA . Drift of the subgingival periodontal microbiome during chronic periodontitis in type 2 diabetes mellitus patients. Pathogens. 2021;10:504. 33922308 10.3390/pathogens10050504 PMC8145315

[34] Ahmadi S Wang S Nagpal R Wang B Jain S Razazan A . A human-origin probiotic cocktail ameliorates aging-related leaky gut and inflammation via modulating the microbiota/taurine/tight junction axis. JCI Insight. 2020;5:e132055. 32302292 10.1172/jci.insight.132055 PMC7253024

[35] Hou Q Huang Y Wang Y Liao L Zhu Z Zhang W . Lactobacillus casei LC01 regulates intestinal epithelial permeability through miR-144 targeting of OCLN and ZO1. J Microbiol Biotechnol. 2020;30:1480–7. 32807750 10.4014/jmb.2002.02059 PMC9728266

[36] Zhang W Li H Zhao N Luo X Liu S Bao a . Lactobacillus johnsonii BS15 combined with abdominal massage on intestinal permeability in rats with nonalcoholic fatty liver and cell biofilm repair. Bioengineered. 2021;12:6354–6363. 34511035 10.1080/21655979.2021.1954134 PMC8806615

[37] Al-Sadi R Dharmaprakash V Nighot P Guo S Nighot M Do T . Bifidobacterium bifidum enhances the intestinal epithelial tight junction barrier and protects against intestinal inflammation by targeting the toll-like receptor-2 pathway in an NF-κB-independent manner. Int J Mol Sci. 2021;22:8070. 34360835 10.3390/ijms22158070 PMC8347470

[38] Hsieh CY Osaka T Moriyama E Date Y Kikuchi J Tsuneda S . Strengthening of the intestinal epithelial tight junction by Bifidobacterium bifidum. Physiol Rep. 2015;3:e12327. 25780093 10.14814/phy2.12327 PMC4393161

[39] Ling X Linglong P Weixia D Hong W . Protective effects of Bifidobacterium on intestinal barrier function in LPS-induced enterocyte barrier injury of Caco-2 monolayers and in a rat NEC model. PLoS One. 2016;11:e0161635. 27551722 10.1371/journal.pone.0161635 PMC4995054

[40] Zoetendal EG Raes J van den Bogert B Arumugam M Booijink CC Troost FJ . The human small intestinal microbiota is driven by rapid uptake and conversion of simple carbohydrates. Isme j. 2012;6:1415–26. 22258098 10.1038/ismej.2011.212 PMC3379644

[41] Neis EP van Eijk HM Lenaerts K Olde Damink SW Blaak EE Dejong CH . Distal versus proximal intestinal short-chain fatty acid release in man, in. Gut. 2019;68:764–765. 29618497 10.1136/gutjnl-2018-316161

[42] Akiba Y Inoue T Kaji I Higashiyama M Narimatsu K Iwamoto K . Short-chain fatty acid sensing in rat duodenum. J Physiol. 2015;593:585–99. 25433076 10.1113/jphysiol.2014.280792 PMC4324707

[43] Wan Saudi WS Sjöblom M . Short-chain fatty acids augment rat duodenal mucosal barrier function. Exp Physiol. 2017;102:791–803. 28436589 10.1113/EP086110

