# Supplementary material for: Regional changes in intestinal permeability in cirrhosis are associated with mucosal bacteria
Source: Hepatol Commun. 2023 Sep 27;7(10):e0221. doi: 10.1097/HC9.0000000000000221 (PMC10531369; doi:10.1097/HC9.0000000000000221)
Supplement: Supplementary file 1 [file hc9-7-e0221-s001.docx]

**Supplementary Table 1: Samples Collected**

|  | **Cirrhosis**  **(N = 58 patients)** | **Controls**  **(N = 33 patients)** |
| --- | --- | --- |
| **Samples for TEER** | | |
| Duodenum biopsy | 44 | 21 |
| Ileum biopsy | 13 | 12 |
| Colon biopsy | 18 | 14 |
| **Total** | **75** | **47** |
| **Samples for 16S rRNA sequencing** | | |
| Duodenal aspirate | 38 | 8 |
| Duodenum biopsy | 49 | 10 |
| Ileum biopsy | 16 | 14 |
| Colon biopsy | 20 | 16 |
| **Total Mucosal Biopsies** | **85** | **40** |
