# Supplementary material for: Regional changes in intestinal permeability in cirrhosis are associated with mucosal bacteria
Source: Hepatol Commun. 2023 Sep 27;7(10):e0221. doi: 10.1097/HC9.0000000000000221 (PMC10531369; doi:10.1097/HC9.0000000000000221)
Supplement: Supplementary file 2 [file hc9-7-e0221-s002.docx]

**Supplementary Table 2: TEER Comparisons by Markers of Cirrhosis Severity**

|  | **High Severity** | **Low Severity** | ***P* value** |
| --- | --- | --- | --- |
| **History of Ascites** | **Ascites** | **No Ascites** |  |
| All segments | (n=40)  12.9 (4.5) | (n=34)  12.6 (4.3) | 0.73 |
| Duodenum | (n=22)  13.9 (5.3) | (n=21)  13.6 (4.8) | 0.95 |
| **History of Hepatic Encephalopathy** | **Hepatic Encephalopathy** | **No Hepatic Encephalopathy** |  |
| All segments | (n=19)  13.9 (4.6) | (n=56)  12.4 (4.2) | 0.15 |
| Duodenum | (n=12)  15.5 (5.1) | (n=32)  13.0 (4.8) | 0.10 |
| **MELD^1^** | **MELD > 10** | **MELD < 10** |  |
| All segments | (n=30)  13.3 (5.0) | (n=44)  12.4 (4.0) | 0.63 |
| Duodenum | (n=16)  14.8 (6.1) | (n=27)  13.2 (4.2) | 0.59 |
| **Platelet Count^2^** | **Platelets < 129/nL** | **Platelets > 129/nL** |  |
| All segments | (n=37)  13.9 (5.2) | (n=38)  11.6 (3.0) | 0.10 |
| Duodenum | (n=23)  15.5 (5.5) | (n=21)  11.7 (3.3) | **0.02** |

*MELD model for end-stage liver disease*

*^1^ The 75% quartile for the cohort were selected as the break point, and patients were compared above and below that 75% quartile. Median was not used as MELD of 8 (median) is a low value.*

*^2^ The median for the cohort were selected as the break point, and patients were compared above and below the median value.*
