# Supplementary material for: Regional changes in intestinal permeability in cirrhosis are associated with mucosal bacteria
Source: Hepatol Commun. 2023 Sep 27;7(10):e0221. doi: 10.1097/HC9.0000000000000221 (PMC10531369; doi:10.1097/HC9.0000000000000221)
Supplement: Supplementary file 3 [file hc9-7-e0221-s003.docx]

**Supplementary Table 3: Comparison of Bacteria Composition between Patients with Cirrhosis and Controls**

|  | ***P* value from AMOVA** | **# Taxa that differ between groups in LEfSe** |
| --- | --- | --- |
| Duodenum, ileum, colon mucosa combined | **< 0.001** | 40 |
| Duodenum mucosa | 0.156 | 5 |
| Ileum mucosa | 0.314 | 26 |
| Colon mucosa | 0.067 | 30 |
| Duodenum aspirate | **0.011** | 12 |
